# Supplementary material for: Upregulation of OASIS/CREB3L1 in podocytes contributes to the disturbance of kidney homeostasis
Source: Commun Biol. 2022 Jul 22;5:734. doi: 10.1038/s42003-022-03709-x (PMC9307819; doi:10.1038/s42003-022-03709-x)
Supplement: Supplementary file 2 — Supplementary Information [file 42003_2022_3709_MOESM2_ESM.pdf]

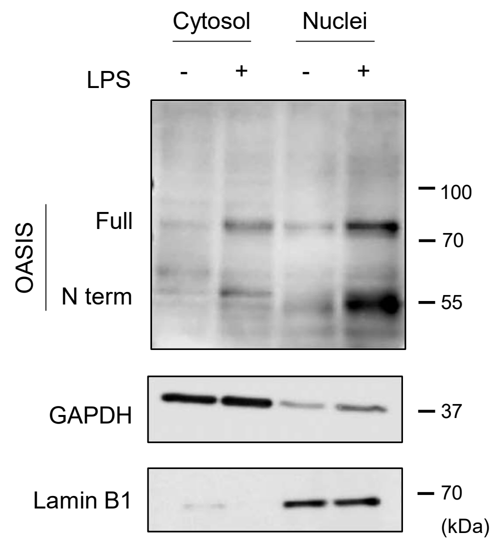

Supplementary Figure 1. ***The N-terminal domain of OASIS was increased in the nuclear fraction of LPS-treated podocytes.***

Podocytes were stimulated with LPS (10 ng/mL) for 6 h. The cytosolic and nuclear fraction were prepared, and immunoblotting was performed using anti-OASIS, anti-Lamin B1 and anti-GAPDH antibodies. Representative images are shown.

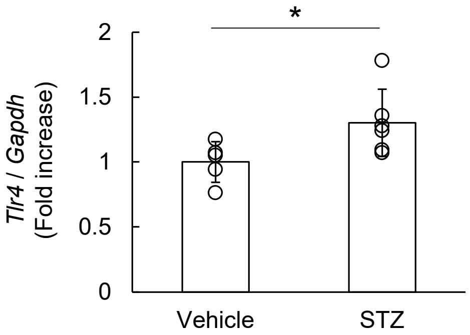

Supplementary Figure 2. ***Tlr4 mRNA expression level was higher in STZ-induced DN model.***

C57BL/6 mice were intraperitoneally administered with STZ. The transcript expression level of *Tlr4* was examined at 8-12 weeks after STZ treatment, using quantitative PCR. The expression level was normalized to that of *Gapdh*. Data are shown as mean ± SD (n=5 for vehicle and n=6 for STZ), $^{*}P < 0.05$, as analyzed using Student's *t*-test.

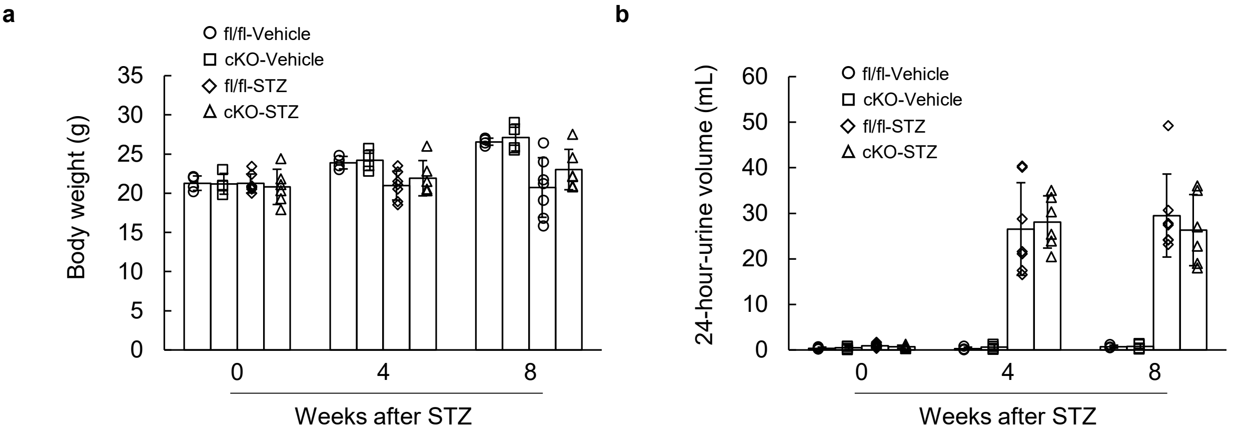

Supplementary Figure 3. ***There were no differences in body weight and urine volume between OASIS cKO and fl/fl mice in STZ-induced DN model.***

a-b OASIS cKO and fl/fl mice were subjected to uninephrectomy, followed by treatment with STZ or vehicle, as a control. Body weight and twenty-four-hour-urine volume were measured at 0, 4 and 8 weeks after STZ treatment. Data are shown as mean ± SD (n=4 for fl/fl-vehicle and cKO-vehicle, n=7 for fl/fl-STZ, and n=6 for cKO-STZ).

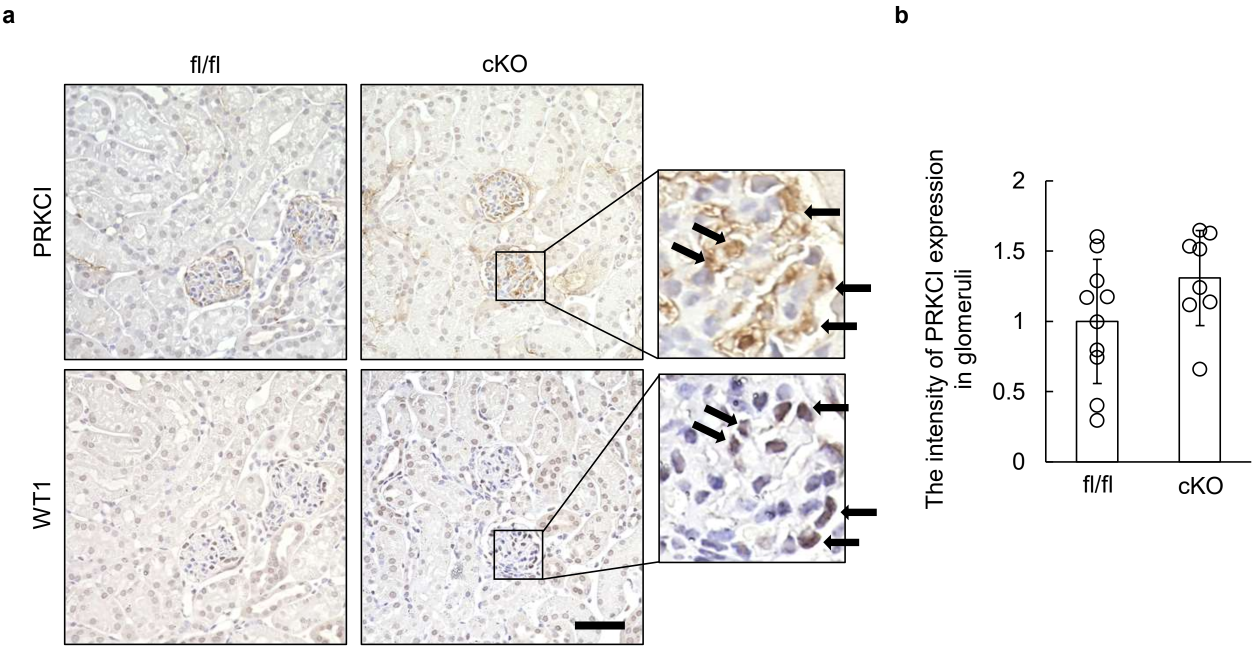

Supplementary Figure 4. ***The PRKCI protein expression in glomeruli was not significantly altered in OASIS cKO mice.***

Immunohistochemistry was performed using an anti-PRKCI or anti-WT-1 antibody in serial sections of the LPS-treated fl/fl and cKO mice. Representative images and the intensity of PRKCI expression in glomeruli are shown. Arrows: PRKCI expressed in podocytes. Scale bar: 50 µm. Data are shown as mean ± SD (n=10 for fl/fl-LPS and n=8 for cKO-LPS).

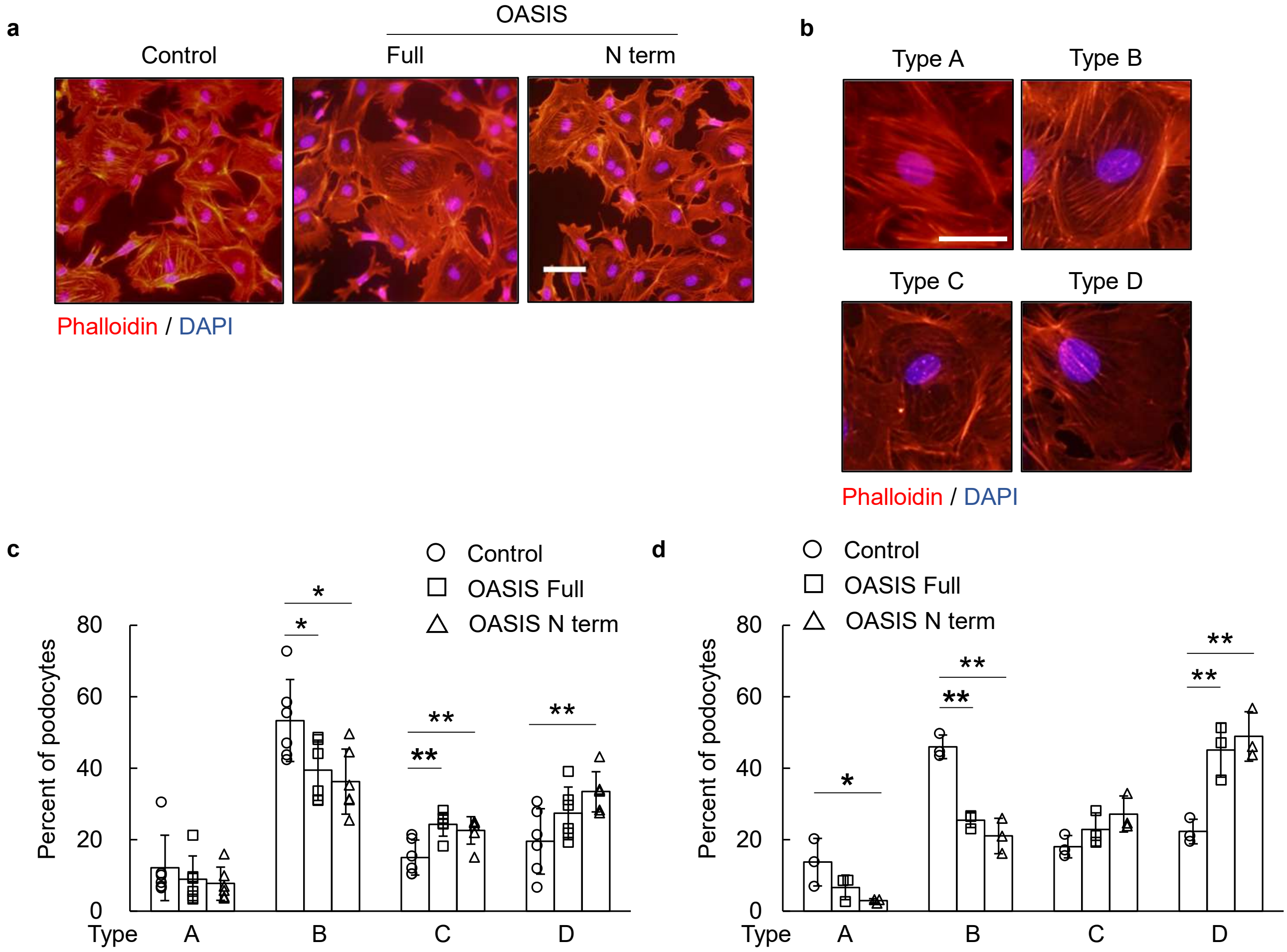

Supplementary Figure 5. ***Actin stress fibers were reduced in OASIS-overexpressed podocytes.***

a-c Murine cultured podocytes were transfected with a lentivirus expressing the full length or active form of *Oasis*, or *venus* (as a control). Two or four days after transfection, immunofluorescence analysis was performed using Alexa Fluor 546-phalloidin and DAPI. **a** Representative images of podocytes treated with lentiviruses for 4 days are shown. Scale bar: 20 μm. **b** Representative images of different types of phalloidin staining patterns are shown. Scale bar: 10 μm. **c-d** Quantification of phalloidin staining patterns in podocytes was performed 2 (**c**) or 4 (**d**) days after transfection of lentiviruses. Data are shown as mean ± SD (n=6 (**c**), n=3 (**d**) for each group), $^{*}P < 0.05$ and $^{**}P < 0.01$, as analyzed using Dunnett test.

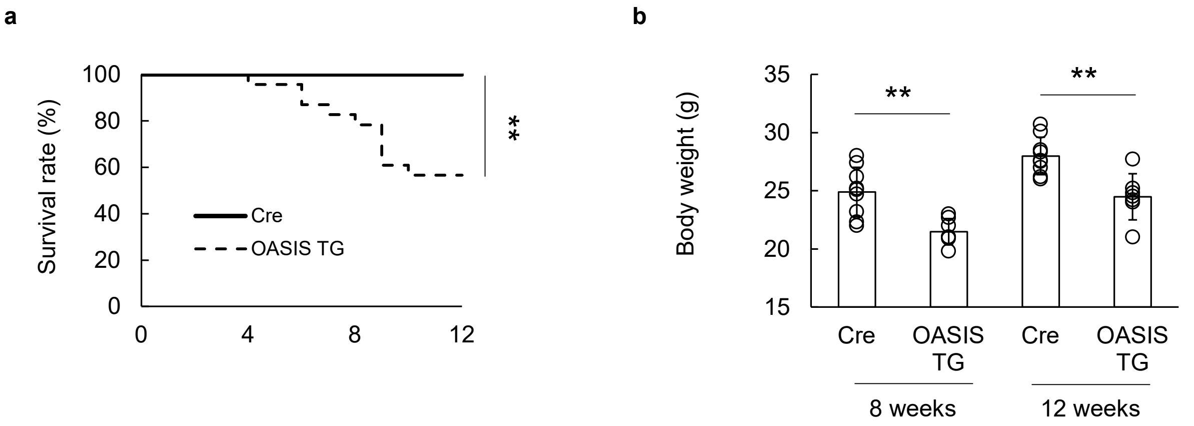

Supplementary Figure 6. ***Survival rate and body weight were decreased in mice overexpressing the podocyte-restricted OASIS.***

a The survival rate after birth was estimated using the Kaplan-Meier method and log-rank test (n=21 for Cre and n=23 for OASIS TG). $^{**}P< 0.01$. **b** Body weight was measured in Cre or OASIS TG mice. Data are shown as mean ± SD (n=9 for Cre and n=7 for OASIS TG), $^{**}P< 0.01$, as analyzed using Student's *t*-test at each time-point.

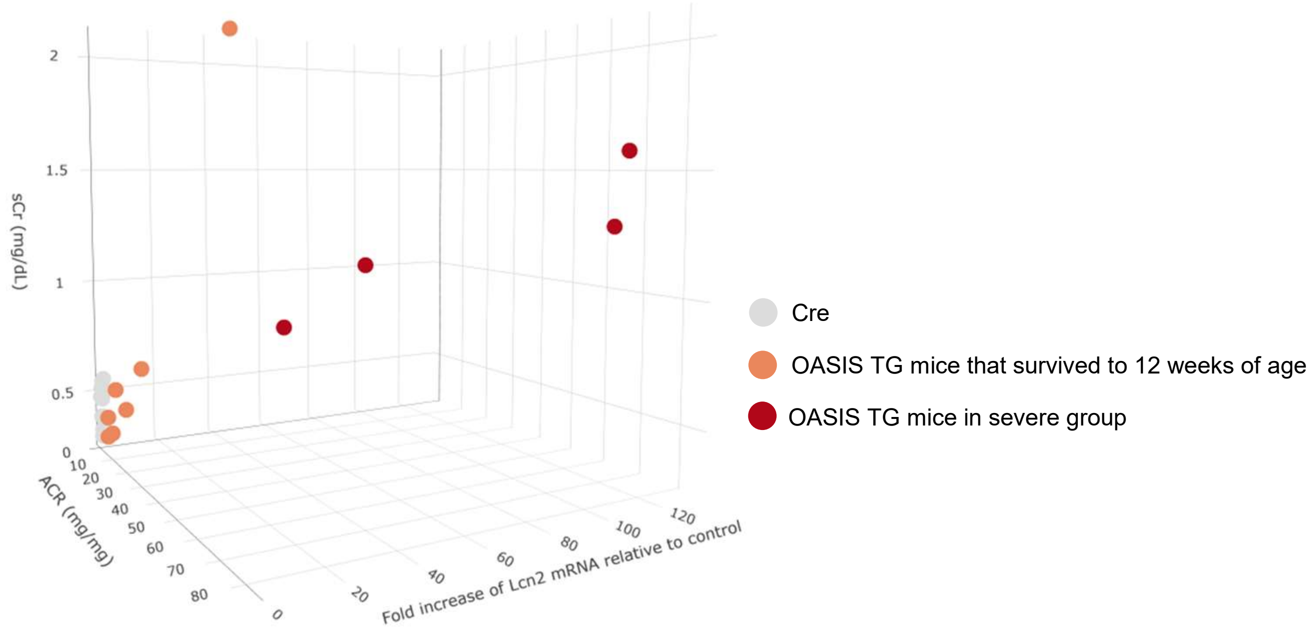

Supplementary Figure 7. ***Kidney function was decreased in severe group of OASIS TG mice.***

sCr and the expression of *Lcn2* mRNA was examined in OASIS TG mice with ACR levels exceeding 15,000 μg/mg by the age of 8 weeks. (n=9 for Cre, n=7 for OASIS TG mice that survived to 12 weeks of age, and n=4 for OASIS TG mice in severe group).

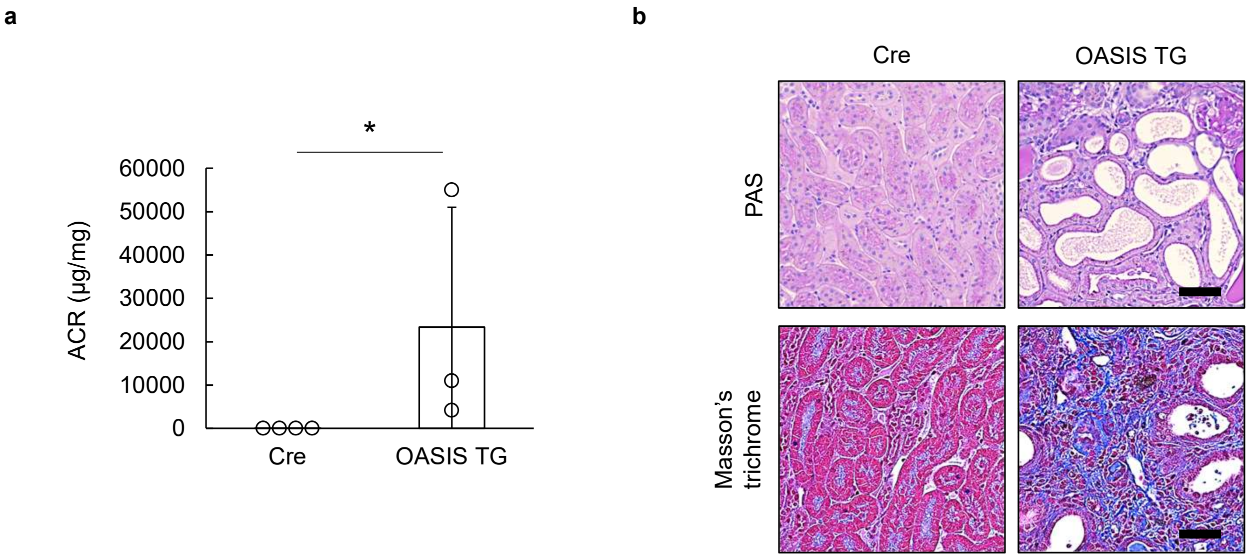

Supplementary Figure 8. ***Kidney injury was also observed in another line of podocyte-restricted OASIS TG mice.*** ACR, and representative images of Periodic acid-Schiff staining and Masson's trichrome staining were shown. Scale bar: 50 µm. Data are shown as mean ± SD (n=4 for Cre and n=3 for OASIS TG), **P*<0 .05, as analyzed using Mann–Whitney U test.

Supplementary Figure 9. ***Unedited blot/gel images.***

Figure 1D

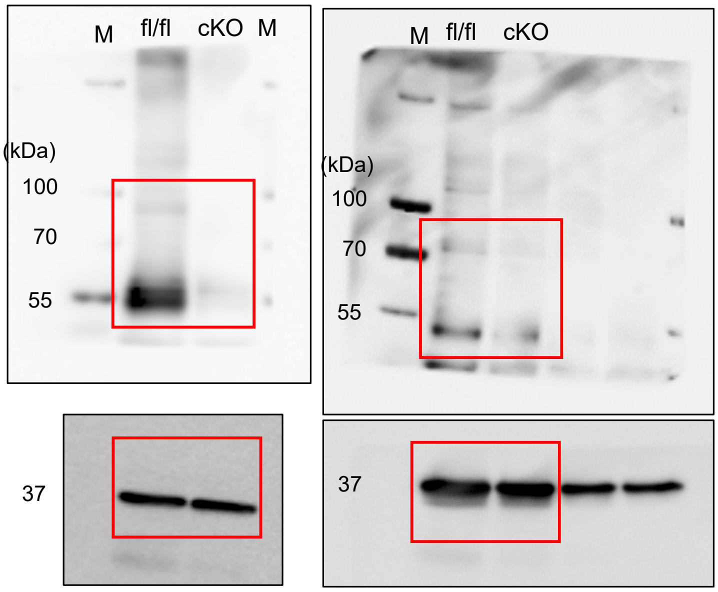

Figure 1E

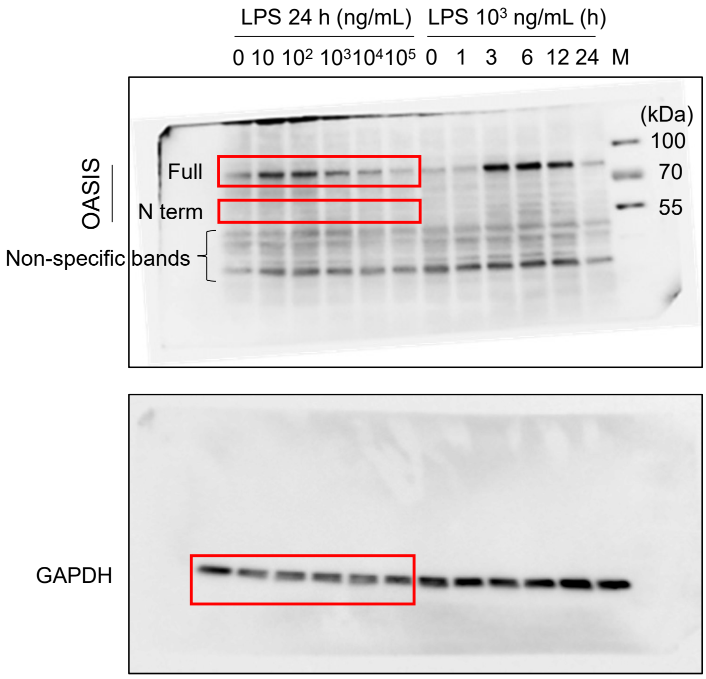

M: molecular marker

Figure 1F

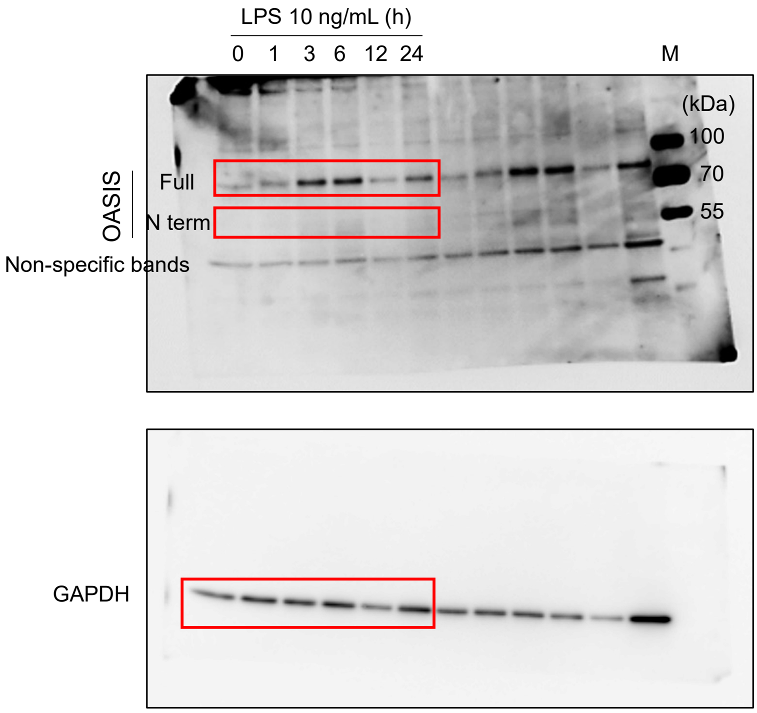

M: molecular marker

Supplementary Figure 9. ***Unedited blot/gel images.***

Figure 1G

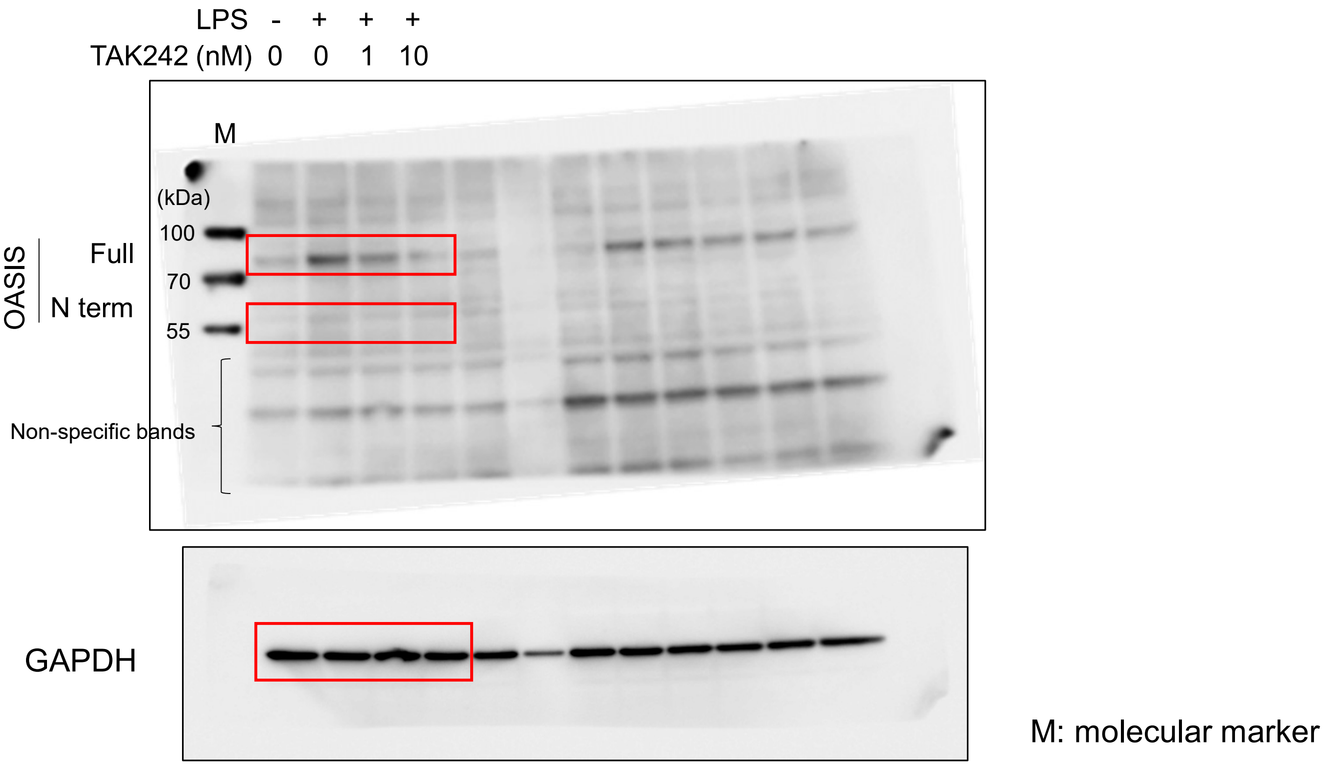

Figure 1H

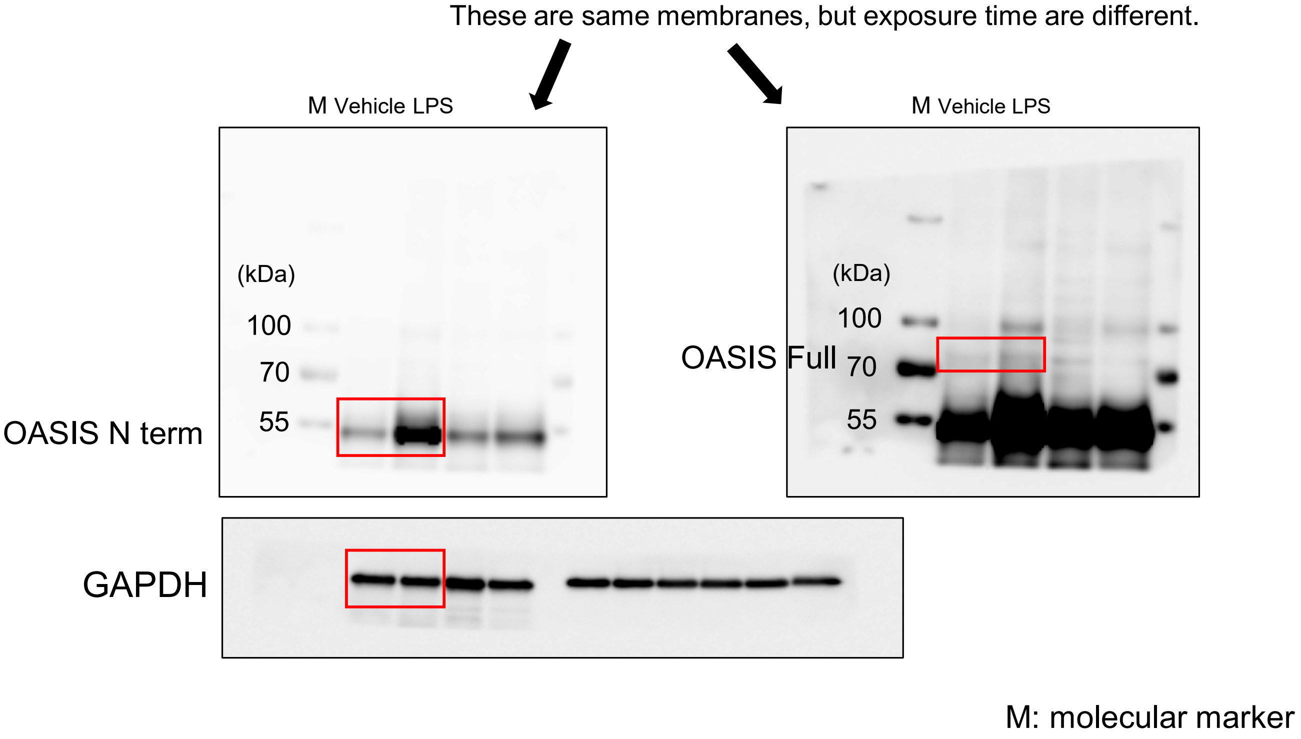

Supplementary Figure 9. ***Unedited blot/gel images.***

Figure 4A

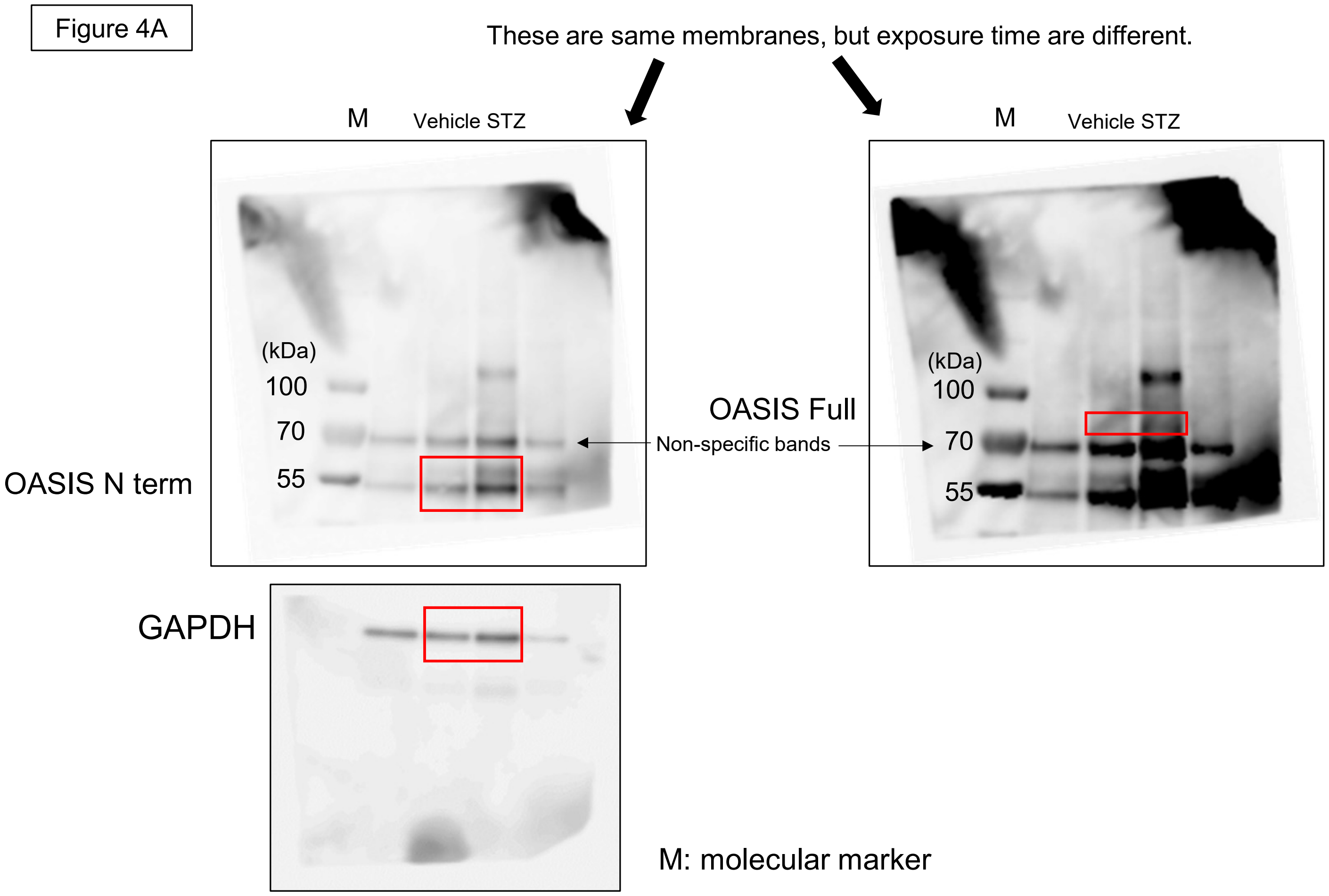

Figure 5A

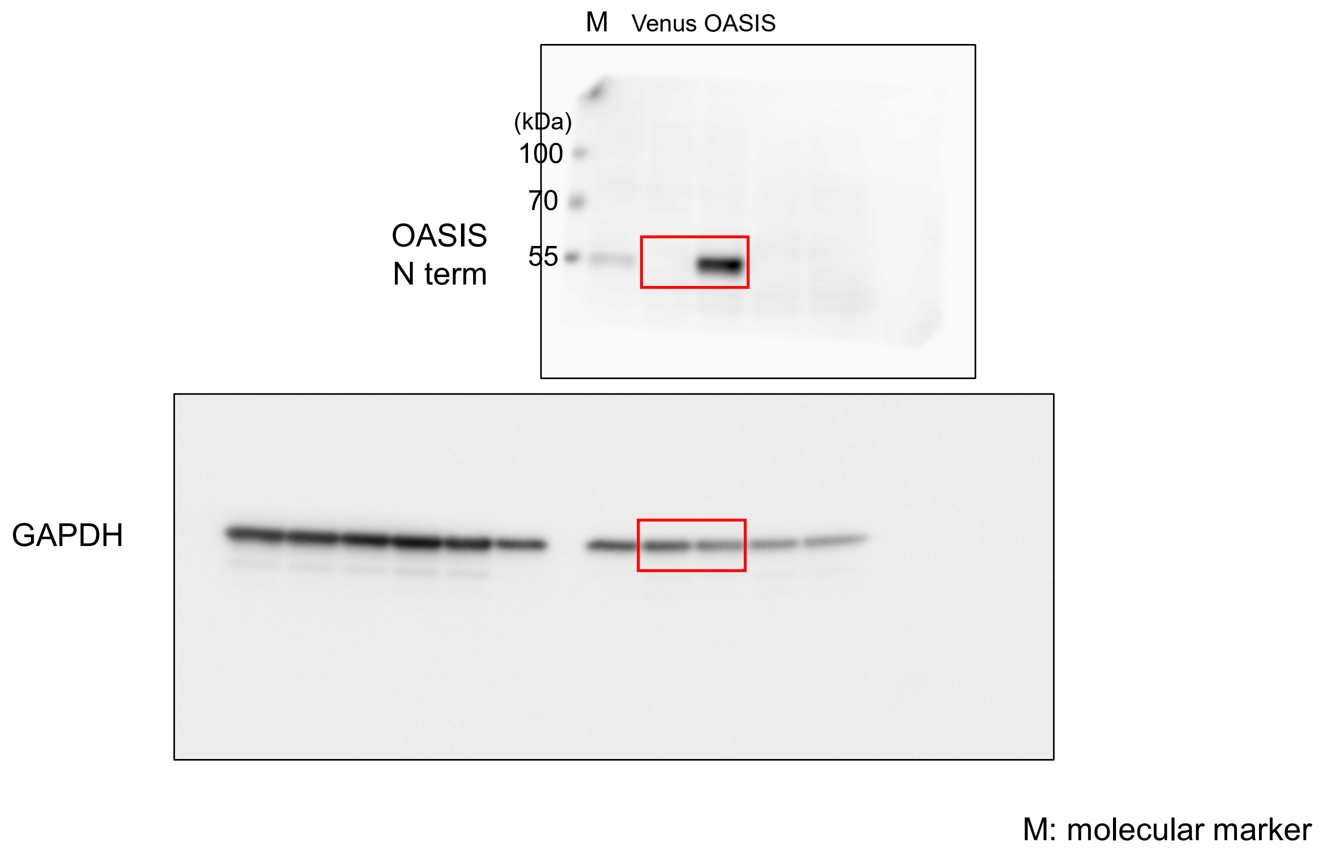

Supplementary Figure 9. *Unedited blot/gel images.*

Figure 5G

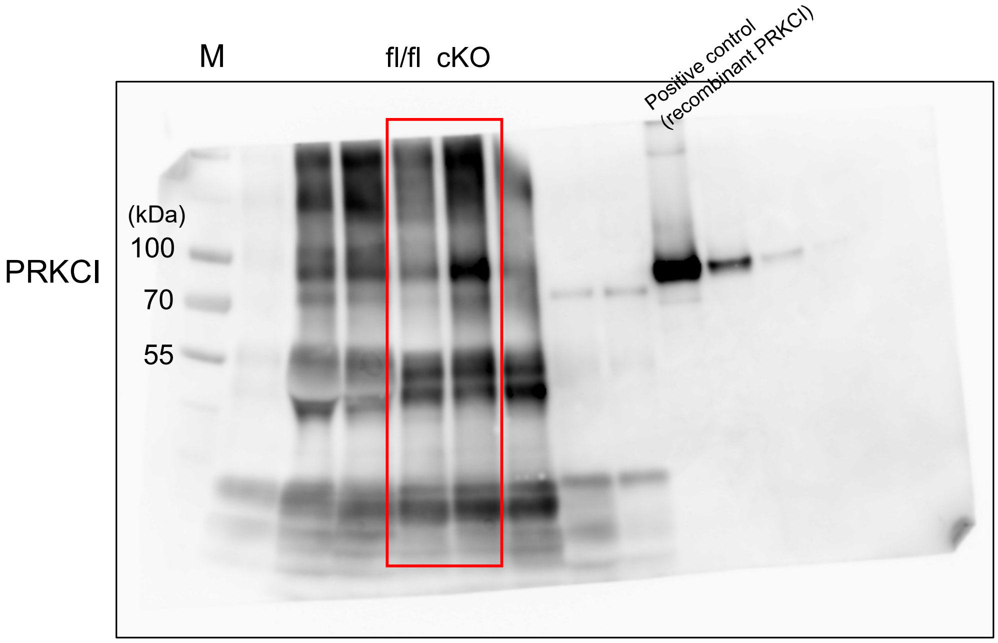

M: molecular marker

Figure 6B

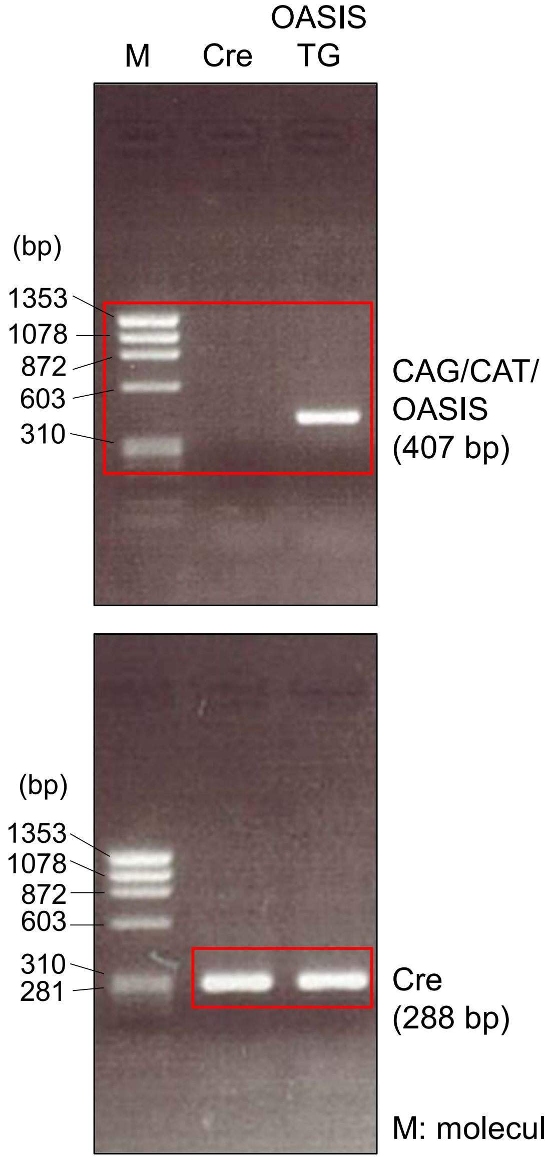

M: molecular marker

Figure 6C

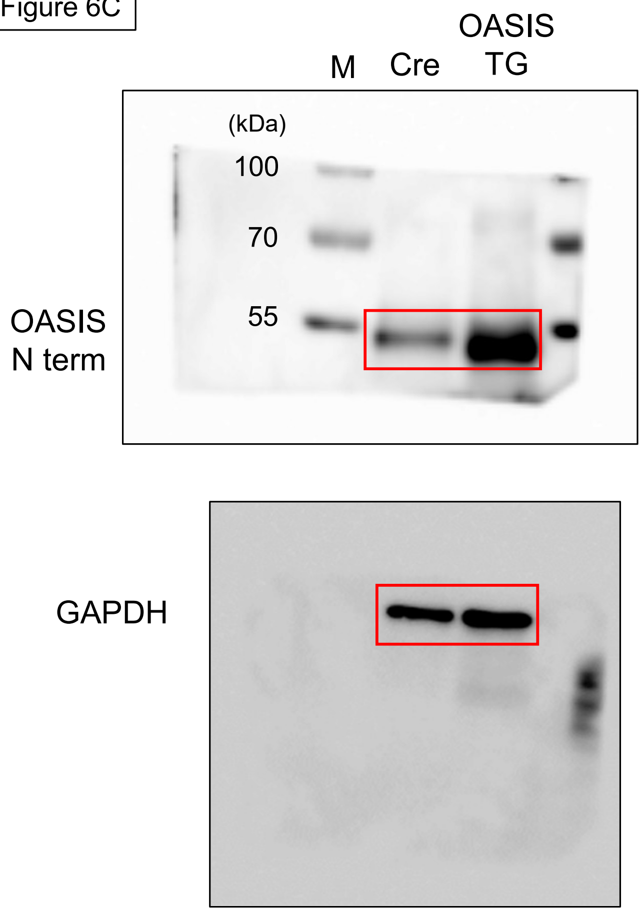

M: molecular marker

Supplementary Figure 9. ***Unedited blot/gel images.***

Supplementary Figure 1

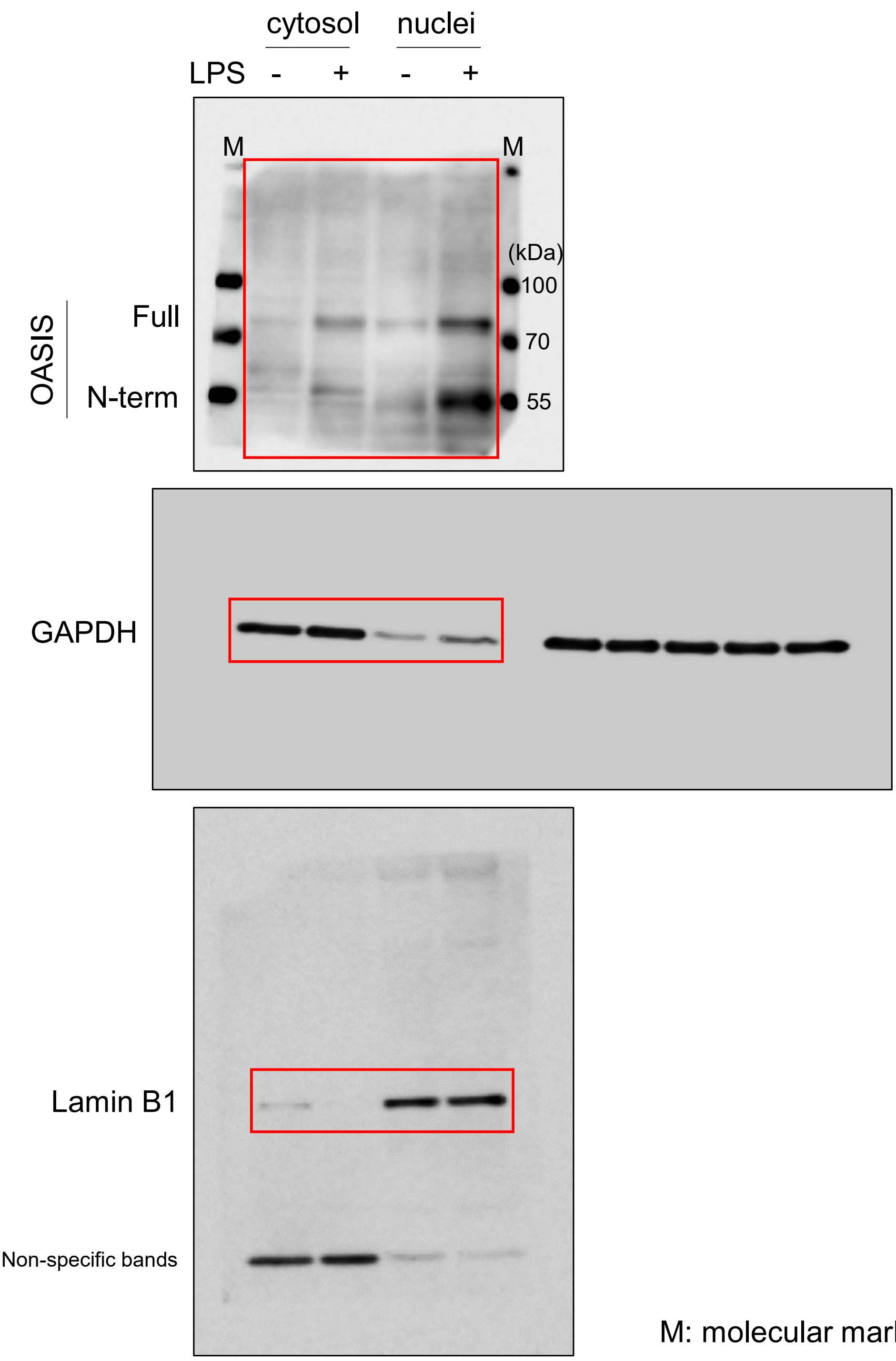

M: molecular marker

Supplementary Tables

Supplementary Table 1. The primers used in this study.

Gene	Species	Direction	Sequence	Usage
Gapdh	Mouse	Forward	5'-CATCACCATCTTCCAGGAGCG-3'	qPCR
		Reverse	5'-GAGGGGCCATCCACAGTCTTC-3'	
Nphs1		Forward	5'-CCCAGAAGCTGTGGATTGAG-3'	
		Reverse	5'-GAAAGTGCTGCCGGACTTCT-3'	
Nphs2		Forward	5'-CATCAAGCCCTCTGGATTAG-3'	
		Reverse	5'-CAGATGTCCCAGTCGGAATA-3'	
Lcn2		Forward	5'-GGCTGTCGCTACTGGATCAG-3'	
		Reverse	5'-GTCCGTGGTGGCCACTTGCA-3'	
Col1a1		Forward	5'-GGCAAGAATGGAGATGATGG-3'	
		Reverse	5'-ATCCAAACCACTGAAGCCTC-3'	
Fibronectin		Forward	5'-ACACCATACCTGCCGAATGTAG-3'	
		Reverse	5'-GAGAGCTTCCTGTCCTGTAGAG-3'	
Prkci		Forward	5'-GCAGTGAGGTTCGAGATATG-3'	
		Reverse	5'-GCCTGAAAGCCTCTTCTAAC-3'	
Wnt5a		Forward	5'-CAGTACCAGTTCCGGCATCG-3'	
		Reverse	5'-GCTCGGCTCATGGCGTTCAC-3'	
Edpr1		Forward	5'-TGTCCTACGATGGTCTCAAC-3'	
		Reverse	5'-AAGGGGATCTTTGCACACTG-3'	
Gadd45 α		Forward	5'-CTGCTACTGGAGAACGAC-3'	
		Reverse	5'-GATCCATGTAGCGACTTTCC-3'	
Rdh11		Forward	5'-GATCCACTTCCATAACCTGC-3'	
		Reverse	5'-TAATAGAGGAGTACCGCGTC-3'	
Pten		Forward	5'-TTGAGTTCCCTCAGCCATTG-3'	
		Reverse	5'-TTCTGAGGTTTCCTCTGGTC-3'	
Cdc37l1		Forward	5'-AGGAGAGTGACTTAGACGTG-3'	
		Reverse	5'-CTAAGCTGCCAAGTTTCTGC-3'	
Adamts12		Forward	5'-CACATGAACTTGGTCACAGC-3'	
		Reverse	5'-AGAAGCGAGTGATGTACTCC-3'	
Maged2		Forward	5'-CTGGGATTCATCACTCACTC-3'	
		Reverse	5'-AAGAACTCGTACTCAGGAGG-3'	

(continued)

Gene	Species	Direction	Sequence	Usage
Polr3k	Mouse	Forward	5’-GCTAAAAGAAGTGGACGACG-3’	qPCR
		Reverse	5’-TAGAAGGTGGTCATTGGCTC-3’	
Cgref1		Forward	5’-GCTTTCGCTCTTTGCTCTTC-3’	
		Reverse	5’-AGCACCGAGTCTACTACTAG-3’	
Col7a1		Forward	5’-GTACCAAGTATGGCTGACTG-3’	
		Reverse	5’-CAATAGAGGTATCCACCACG-3’	
Ada		Forward	5’-CCATCAAGCCAGAAACCATC-3’	
		Reverse	5’-GTCCATGCCGATAATGTTGC-3’	
Pcolce2		Forward	5’-AGCAGAAGTGTAGACGGATG-3’	
		Reverse	5’-ACACTCATGTTCTTGCCAGC-3’	
Metrnl		Forward	5’-CCAGTATGAGCTGATGAGTG-3’	
		Reverse	5’-GGTAGATGACTGACACTTGC-3’	
Col1a2		Forward	5’-TCTCTACTGGTGAAACCTGC-3’	
		Reverse	5’-GCTGAGTTGCCATTTCCTTG-3’	
Mmel1		Forward	5’-CTCCAGATACAGCGTCTTTG-3’	
		Reverse	5’-GGAGCGATATAGTGTCTTGG-3’	
Adamts17		Forward	5’-ACATGTACCAACTCCCAAGG-3’	
		Reverse	5’-TCCAGTGAGCTTGTGCATAC-3’	
Hpse		Forward	5’-GAGAGCAGTACCAAAAGGAG-3’	
		Reverse	5’-CAGTAGTCAAGGAGAAGCTG-3’	
Dusp13		Forward	5’-ATGGATCTTCCCCAATCGTG-3’	
		Reverse	5’-AGAGTAGCAAACTGGCAGAG-3’	
Lox		Forward	5’-AGGGTACTGCTACGATTTCC-3’	
		Reverse	5’-GGTCATAGTGGCTGAATTCG-3’	
Tlr4		Forward	5’-GAAACTCAGCAAAGTCCCTG-3’	
		Reverse	5’-GTTTGAGAGGTGGTGTAAGC-3’	
Lcn2	Human	Forward	5’-TGTGACTACTGGATCAGGAC-3’	
		Reverse	5’-GGGTGATCTTGAAGTACTCC-3’	
Oasis/Creb3l1	Mouse	Forward	5’-GCTGGACGAGAAGAGTGCTCTG-3’	RT-PCR
		Reverse	5’-GTTCCACATCTTGAGTGGTGTC-3’	

.

(continued)

Gene	Species	Direction	Sequence	Usage
Cre recombinase	Mouse	Forward	5'-CGATGCAACGAGTGATGAGG-3'	Genotyping
		Reverse	5'-GCATTGCTGTCACTTGGTCGT-3'	
Oasis flox		Forward	5'-AAGGTGAGGAAGTTTGTGAGATTG-3'	
		Reverse	5'-TAAGTGGGGTTGGAGGATCTAAATC-3'	
Oasis TG		Forward	5'-GCCATACCACATTTGTAGAG-3'	
		Reverse	5'-TGAGGAAATCCGACTCATTC-3'	
