# Supplementary material for: Upregulation of OASIS/CREB3L1 in podocytes contributes to the disturbance of kidney homeostasis
Source: Commun Biol. 2022 Jul 22;5:734. doi: 10.1038/s42003-022-03709-x (PMC9307819; doi:10.1038/s42003-022-03709-x)
Supplement: Supplementary file 5 — Reporting Summary [file 42003_2022_3709_MOESM5_ESM.pdf]

nature portfolio

Corresponding author(s): Masanori Obana

Last updated by author(s): Jun 20, 2022

Reporting Summary

Nature Portfolio wishes to improve the reproducibility of the work that we publish. This form provides structure for consistency and transparency in reporting. For further information on Nature Portfolio policies, see our Editorial Policies and the Editorial Policy Checklist.

Statistics

For all statistical analyses, confirm that the following items are present in the figure legend, table legend, main text, or Methods section.

n/a	Confirmed	
☐	☒	The exact sample size (n) for each experimental group/condition, given as a discrete number and unit of measurement
☐	☒	A statement on whether measurements were taken from distinct samples or whether the same sample was measured repeatedly
☐	☒	The statistical test(s) used AND whether they are one- or two-sided<br>*Only common tests should be described solely by name; describe more complex techniques in the Methods section.*
☐	☒	A description of all covariates tested
☐	☒	A description of any assumptions or corrections, such as tests of normality and adjustment for multiple comparisons
☐	☒	A full description of the statistical parameters including central tendency (e.g. means) or other basic estimates (e.g. regression coefficient) AND variation (e.g. standard deviation) or associated estimates of uncertainty (e.g. confidence intervals)
☐	☒	For null hypothesis testing, the test statistic (e.g. F, t, r) with confidence intervals, effect sizes, degrees of freedom and P value noted<br>*Give P values as exact values whenever suitable.*
☒	☐	For Bayesian analysis, information on the choice of priors and Markov chain Monte Carlo settings
☒	☐	For hierarchical and complex designs, identification of the appropriate level for tests and full reporting of outcomes
☒	☐	Estimates of effect sizes (e.g. Cohen's d, Pearson's r), indicating how they were calculated

Our web collection on statistics for biologists contains articles on many of the points above.

Software and code

Policy information about availability of computer code

Data collection	No software was used.
Data analysis	The analyses were performed using Statcel4.

For manuscripts utilizing custom algorithms or software that are central to the research but not yet described in published literature, software must be made available to editors and reviewers. We strongly encourage code deposition in a community repository (e.g. GitHub). See the Nature Portfolio guidelines for submitting code & software for further information.

Data

Policy information about availability of data

All manuscripts must include a data availability statement. This statement should provide the following information, where applicable:

- Accession codes, unique identifiers, or web links for publicly available datasets
- A description of any restrictions on data availability
- For clinical datasets or third party data, please ensure that the statement adheres to our policy

The data that support the findings of this study are available from the corresponding author, M.O., upon reasonable request.

Field-specific reporting

Please select the one below that is the best fit for your research. If you are not sure, read the appropriate sections before making your selection.

☒ Life sciences ☐ Behavioural & social sciences ☐ Ecological, evolutionary & environmental sciences

For a reference copy of the document with all sections, see nature.com/documents/nr-reporting-summary-flat.pdf

Life sciences study design

All studies must disclose on these points even when the disclosure is negative.

Sample size	The sample size was chosen based on previous report (Nat Commun. 2019;10(1):4523., J Am Soc Nephrol. 2016;27(9):2685-700.) and our preliminary experiments.
Data exclusions	No data were excluded from the analyses.
Replication	The reproducibility of all experiments was confirmed.
Randomization	To perform the histological evaluation, the histological images were randomly taken.
Blinding	Quantification was carried out by a researcher who was blinded to the experimental groups.

Reporting for specific materials, systems and methods

We require information from authors about some types of materials, experimental systems and methods used in many studies. Here, indicate whether each material, system or method listed is relevant to your study. If you are not sure if a list item applies to your research, read the appropriate section before selecting a response.

Materials & experimental systems

n/a	Involved in the study	
☐	☒	Antibodies
☐	☒	Eukaryotic cell lines
☒	☐	Palaeontology and archaeology
☐	☒	Animals and other organisms
☐	☒	Human research participants
☒	☐	Clinical data
☒	☐	Dual use research of concern

Methods

n/a	Involved in the study	
☒	☐	ChIP-seq
☒	☐	Flow cytometry
☒	☐	MRI-based neuroimaging

Antibodies

Antibodies used	Anti-OASIS (MABE1017; Millipore), anti-OASIS (AF4080; R&D systems), anti-WT-1 (sc-393498; Santa Cruz Biotechnology), anti-Lamin B1 (3C10G12; Proteintech), anti-PRKCI (610175; BD Biosciences), and anti-GAPDH (MAB374; Millipore) antibodies were used in this study.
Validation	The validation of anti-OASIS (MABE1017; Millipore) and anti-OASIS (AF4080; R&D systems) antibodies was performed in our previous study (FASEB J. 2021;35(2):e21158.). The validation of anti-PRKCI antibody was performed in this study (data not shown). The antibodies against WT-1, Lamin B1, and anti-GAPDH are widely used worldwide.

Eukaryotic cell lines

Policy information about cell lines

Cell line source(s)	Immortalized murine podocyte cell line was obtained from Dr. Mundel P.
Authentication	The cell lines used in this study are widely used worldwide (J Am Soc Nephrol. 2021;32(3):597-613.).
Mycoplasma contamination	We confirmed the cell lines tested negative from mycoplasma contamination.
Commonly misidentified lines (See ICLAC register)	Not applicable.

Animals and other organisms

Policy information about studies involving animals; ARRIVE guidelines recommended for reporting animal research

Laboratory animals	C57BL/6J male mice (8-10-week-old) were purchased from Japan SLC. Neph2-Cre mice and Oasis fl/fl mice were established in previous reports (Nat Med. 2008;14(3):290-8.,FASEB J. 2021;35(2):e21158.). Podocyte-restricted OASIS overexpression mice were produced in this study.
Wild animals	n/a
Field-collected samples	n/a
Ethics oversight	Care of animals was performed according to Osaka University animal care guidelines and RIKEN Regulations for the Animal Experiments. All experimental procedures conformed to the Guide for the Care and Use of Laboratory Animals, Eighth Edition, updated by the US National Research Council Committee in 2011, and were approved by Animal Care and Use Committee in Graduate School of Pharmaceutical Sciences, Osaka University (approved number: Douyaku 28-14, R01-1) and by Institutional Animal Care and Use Committee of RIKEN Kobe Branch.

Note that full information on the approval of the study protocol must also be provided in the manuscript.

Human research participants

Policy information about studies involving human research participants

Population characteristics	Kidney biopsies from human subject with minimal change nephrotic syndrome and diabetic nephropathy were obtained. The normal area of the biopsied carcinoma tissues was used as control.
Recruitment	The study was conducted under informed consent and was approved by the ethics committee on human research of the Chiba University Hospital. Regarding the purchased samples, the analysis was approved by Clinical Research Ethics Review Committee in Graduate School of Pharmaceutical Science, Osaka University .
Ethics oversight	No. 1119 (856) and No. Yakuso30-4

Note that full information on the approval of the study protocol must also be provided in the manuscript.
